# Supplementary material for: AD-AutoGPT: An autonomous GPT for Alzheimer’s disease infodemiology
Source: PLOS Glob Public Health. 2025 May 7;5(5):e0004383. doi: 10.1371/journal.pgph.0004383 (PMC12058166; doi:10.1371/journal.pgph.0004383)
Supplement: Attachment — Submitted filename: AD_AutoGPT___Journal_Review_Rebuttal.pdf [file pgph.0004383.s001.pdf]

Reply to the Reviewers

Re: Manuscript ID [PGPH-D-24-00508] - [EMID:3a9f32e4745dc180]
"AD-AutoGPT: An Autonomous GPT for Alzheimer's Disease Infodemiology"
PLoS

To Editors: We sincerely appreciate the reviewers for their thorough review, valuable feedback, and acknowledgment of our work. We have carefully addressed each question and suggestion raised by the reviewers. Notably, several reviewers have expressed particular concern regarding the quality of the eye-gaze data and the model's reliance on it.

The following are responses to journal reviewers' comments: Regarding conflict of interest statement: We would like to thank the editor for helping us revise the conflict of interest statement to comply with the journal's formatting requirements. We have carefully checked the revised statement and confirmed that there are no issues. Please feel free to let us know if you have any further requirements.

Regarding Figure 3:

(a) We have provided a direct link `https://www.statsilk.com/maps/download-free-shapefile-maps` to the base layer of the map (i.e. the country or region boundary shape) and ensure that this information is included in the legend. We have made corresponding changes in the revised version.

(b) We have also provided a link to the terms of use/license information for the base layer image or vector file. We have ensured that the map base layer used complies with the CC-BY 4.0 license and avoid using any proprietary or copyrighted maps (such as Google Maps, Mapquest). Thanks again to the editor for the reminder. We have ensured that all content meets the publication requirements of the work during revision.

We hope that the above reply can address the reviewers' comments. If there are any further questions or suggestions, we are more than willing to continue to cooperate with the revision.

Reviewer #1, comment #1

This paper introduces AD-AutoGPT, an advanced Large Language Model (LLM) tool designed to collect, process, and analyze data based on users' textual prompts, specifically focusing on understanding complex health narratives related to Alzheimer's disease. While AD-AutoGPT holds significant promise for enhancing our comprehension of AD-related health issues, it's crucial to acknowledge potential risks related to the dissemination of misinformation and its impact on public health. Are there any risk mitigation processes or safeguards employed to prevent the mass generation of health disinformation?

Our response #1.1

Thank you for raising your concerns about the spread of misinformation. We fully recognize that when developing tools like AD-AutoGPT, it is crucial to ensure the accuracy and credibility of information. To this end, we have taken the following measures to reduce the risk of generating false information in the health field:

Restrict information sources: AD-AutoGPT strictly limits information crawling from authoritative websites or designated trusted sources. These sources include validated medical databases, peer-reviewed scientific literature, and reputable medical websites. This strategy ensures that the data processed and analyzed by the tool comes from reliable, high-quality sources, radically reducing the likelihood of generating inaccurate information.

Information anti-counterfeiting certification: We use some tools, like Deid-gpt [1], in Auto-GPT to perform anti-counterfeiting certification on the captured information. These tools use advanced AI technology to identify and filter possible fake data or inaccurate information, further ensuring the credibility of the generated content.

We added one paragraph in Section 3.3 to discuss our strategy to improve the trustworthiness of the results of AD-AutoGPT. Through the above measures, we believe that AD-AutoGPT can effectively reduce the risk of spreading false information and provide users with more accurate and credible Alzheimer's disease-related health information. We appreciate your feedback and are open to more discussions on how to further improve and refine these precautions.

Reviewer #2, comment #1

While I find the tool presented useful, the authors should tone down a bit their claims through the text. There are sentences like: "The initial success of AD-AutoGPT has paved the way for future..."

Our response #2.1

We would like to thank the reviewers for their recognition of our work. We realize that some statements in the paper may be exaggerated, such as the expression "The initial success of AD-AutoGPT has laid the foundation for the future...". We revised this sentence in the first paragraph of the conclusion section and checked the wordings in introduction section and related work section appropriately to ensure that the tone of the paper is more cautious and scientific. Our work represents the pioneering application of AutoGPT in the AD field, as it was the first to do so. With 26 citations to date `https://scholar.google.com/citations?view_op=view_citation&hl=en&user=sfEPWiAAAAAJ&citation_for_view=sfEPWiAAAAAJ:u5HHmVD_uO8C`, including references from reputable conferences and journals like RSNA and IEEE, we believe our research has made a significant contribution to the field.

Reviewer #2, comment #2

The state of the art is not discussed in the introduction. Is there the first disease-specific application of AutoGPT? Are there other AD-AutoGTP already? If so, cite them, explain what's different between your tool and these. If not, the authors should emphasize the reproducibility aspect much more since their work can be reused for other diseases.

Our response #2.2

We acknowledge that the discussion of existing research in the introduction is insufficient. We added a discussion of the existing literature in the revised version, especially regarding the current status of AutoGPT in disease-specific applications. AD-AutoGPT is the first application of applying AutoGPT to Alzheimer's disease, we revised the manuscript and highlighted its reproducibility and scalability, emphasizing the potential for this tool to be easily applied to the study of other diseases in the introduction and conclusion section.

Reviewer #2, comment #3

Related to the last point, I would like to see the code in GitHub with clear instructions and a link to figshare for data/videos. The authors should emphasize how someone can reuse their work for any other indication with the minimum effort.

Our response #2.3

We completely agree with the reviewer on the importance of open source. We attached detailed usage instructions and the environment installation process on Github `https://github.com/levyisthebest/AD-AutoGPT`. At the same time, we have also uploaded data and videos to figshare and provide corresponding links. We have explicitly emphasized in the revised version how our work can be reused in other disease studies with minimal effort.

Reviewer #2, comment #4

The samples size seems small (+200 articles). Why not adding many more? I feel a tool with only 200 articles between 2022 and 2023 is outdated for any research. How do the authors plan to keep the tool up to date? Arent there ways to automatize including more articles to it?

Our response #2.4

We acknowledge the reviewer's concerns regarding the sample size. Our current dataset includes just over 200 articles from 2022 and 2023. This limitation is primarily due to our stringent keyword search rules, which reduced the number of qualifying results. Additionally, at the time of our study, the cost of using GPT-4 was relatively high, and our funding constraints prevented us from gathering a larger dataset. Nevertheless, as a methodology-focused paper, we hope that this sample size is sufficient to demonstrate the validity of our approach. Future researchers can use our tool to explore a broader dataset, which we are confident will yield even more meaningful insights.

References

[1] Zhengliang Liu, Yue Huang, Xiaowei Yu, Lu Zhang, Zihao Wu, Chao Cao, Haixing Dai, Lin Zhao, Yiwei Li, Peng Shu, et al. Deid-gpt: Zero-shot medical text de-identification by gpt-4. *arXiv preprint arXiv:2303.11032*, 2023.
