# Supplementary material for: AD-AutoGPT: An autonomous GPT for Alzheimer’s disease infodemiology
Source: PLOS Glob Public Health. 2025 May 7;5(5):e0004383. doi: 10.1371/journal.pgph.0004383 (PMC12058166; doi:10.1371/journal.pgph.0004383)
Supplement: Attachment — Submitted filename: Letter_to_reviewers.pdf [file pgph.0004383.s002.pdf]

Rebuttal

We sincerely appreciate the reviewers for their thorough review, valuable feedback, and acknowledgment of our work. We have carefully addressed each question and suggestion raised by the reviewers. We noticed that reviewers have expressed their concern regarding the quality of the eye-gaze data and the model's reliance on it.

The following are responses to journal reviewers' comments:

We sincerely appreciate your valuable feedback on our manuscript. We have carefully considered the reviewers' comments and made corresponding revisions accordingly.

For Reviewer2:

1.Regarding Github repository

We would like to thank the reviewers for their recognition of our work. We agree that the current github repository may not have been as clear as it could be. In response, we have updated the README.md to include more detailed explanations of each file's purpose and step-by-step instructions for running the code. We have also clarified the process for updating the latest articles and highlighted the demo video as a tutorial. Furthermore, we have added additional comments throughout the code to help future researchers reproduce and build upon our work.

2. Further details regarding the sample of articles.

We sincerely appreciate the reviewer's insightful feedback regarding the sample size and maintenance of our tool. Our current dataset includes just over 200 articles from 2022 and 2023. This relatively small sample size is largely a result of our stringent keyword search criteria, which narrowed down the pool of eligible articles. Additionally, the high cost of using GPT-4, coupled with our funding limitations at the time, further restricted our ability to expand the dataset. However, as this study primarily aims to showcase the methodology, we believe the existing sample size is sufficient to validate our approach and demonstrate its potential.

To address the reviewer's question about maintaining the tool, we designed it with automation in mind. The tool is equipped to automatically search and collect the latest articles on Alzheimer's disease (AD). In the latest update, we have added detailed documentation in our GitHub repository explaining how to configure the environment,

run the program, and input relevant prompts to trigger automated collection of new content. By following these instructions, researchers can continuously update the dataset to align with their needs. We are confident that this functionality will empower other researchers to explore larger datasets and obtain up-to-date insights efficiently. We also highlighted this point in the **Results** section of the paper using blue font..

Thanks again to the reviewers for their valuable comments. We will take these suggestions seriously and make corresponding adjustments in the revision. Hopefully these responses will address the reviewers' concerns. If you have further questions or suggestions, we are more than happy to continue the discussion.
